# Doubled lifespan and patient‐like pathologies in progeria mice fed high‐fat diet

**DOI:** 10.1111/acel.12852

**Published:** 2018-12-12

**Authors:** Ray Kreienkamp, Cyrielle Billon, Gonzalo Bedia‐Diaz, Carolyn J. Albert, Zacharie Toth, Andrew A. Butler, Sara McBride‐Gagyi, David A. Ford, Angel Baldan, Thomas P. Burris, Susana Gonzalo

**Affiliations:** ^1^ Edward A. Doisy Department of Biochemistry and Molecular Biology St Louis University School of Medicine St Louis Missouri; ^2^ Center for Clinical Pharmacology Washington University School of Medicine and St. Louis College of Pharmacy St Louis Missouri; ^3^ Department of Orthopedic Surgery St Louis University School of Medicine St Louis Missouri; ^4^ Department of Pharmacology and Physiology St Louis University School of Medicine St Louis Missouri

## Abstract

Hutchinson‐Gilford Progeria Syndrome (HGPS) is a devastating premature aging disease. Mouse models have been instrumental for understanding HGPS mechanisms and for testing therapies, which to date have had only marginal benefits in mice and patients. Barriers to developing effective therapies include the unknown etiology of progeria mice early death, seemingly unrelated to the reported atherosclerosis contributing to HGPS patient mortality, and mice not recapitulating the severity of human disease. Here, we show that progeria mice die from starvation and cachexia. Switching progeria mice approaching death from regular diet to high‐fat diet (HFD) rescues early lethality and ameliorates morbidity. Critically, feeding the mice only HFD delays aging and nearly doubles lifespan, which is the greatest lifespan extension recorded in progeria mice. The extended lifespan allows for progeria mice to develop degenerative aging pathologies of a severity that emulates the human disease. We propose that starvation and cachexia greatly influence progeria phenotypes and that nutritional/nutraceutical strategies might help modulate disease progression. Importantly, progeria mice on HFD provide a more clinically relevant animal model to study mechanisms of HGPS pathology and to test therapies.

## 1 INTRODUCTION

Hutchinson‐Gilford Progeria Syndrome (HGPS) is caused by *LMNA* gene mutation and expression of a mutant lamin A protein called progerin, which disrupts nuclear architecture and genome stability and function, promoting cellular senescence (Collins, 2016; Gonzalo & Kreienkamp, 2015; Prokocimer, Barkan, & Gruenbaum, 2013). At the organismal level, progerin unleashes a trove of devastating aging pathologies including alopecia, bone and joint abnormalities, lipodystrophy, cardiovascular disease (CVD), and atherosclerosis (Gonzalo, Kreienkamp, & Askjaer, 2016; Ullrich & Gordon, 2015). The progerin‐expressing *Lmna^G609G/G609G^* mouse (herein G609G) carries the equivalent human mutation in homozygosis and has been widely used for studying the mechanistic bases underlying progeria, as well as the outcomes of various therapeutic avenues (Osorio et al., 2011). Cellular phenotypes include nuclear morphological abnormalities, increased genomic instability, deregulated gene expression, and premature senescence (Gonzalo et al., 2016; Ullrich & Gordon, 2015). G609G mice develop some progeroid features such as growth impairment, lipodystrophy, bone abnormalities, and cardiovascular alterations (Osorio et al., 2011; Villa‐Bellosta et al., 2013). However, these mice die at a very young age (~100 days) of unknown etiology and do not mimic the severity of disease of HGPS patients.

Here, we demonstrate that G609G mice succumb to starvation and cachexia, with mice losing lean mass and white adipose tissue at an early age and exhibiting alterations in energy utilization and expenditure. Switching mice nearing death from regular chow (RC) to high‐fat diet (HFD), with higher caloric content, rescues early lethality and significantly improves organismal decline. Importantly, G609G mice placed on HFD in the first month of life nearly double their lifespan and, during their extended survival, they develop HGPS‐like pathologies including severe alopecia, skeletal dysplasia, and aortic wall stiffening. Collectively, our data demonstrate the importance of caloric intake to G609G mouse phenotype, and suggest that alterations in metabolism, both in humans and mice, play a role in progerin‐induced physical decline. Thus, progeria mice on HFD provide a unique model to identify mechanisms underlying progerin‐induced tissue toxicity and for testing therapies of relevance for human disease.

## 2 RESULTS

### 2.1 Progeria mice exhibit metabolic alterations

**Figure 1:**
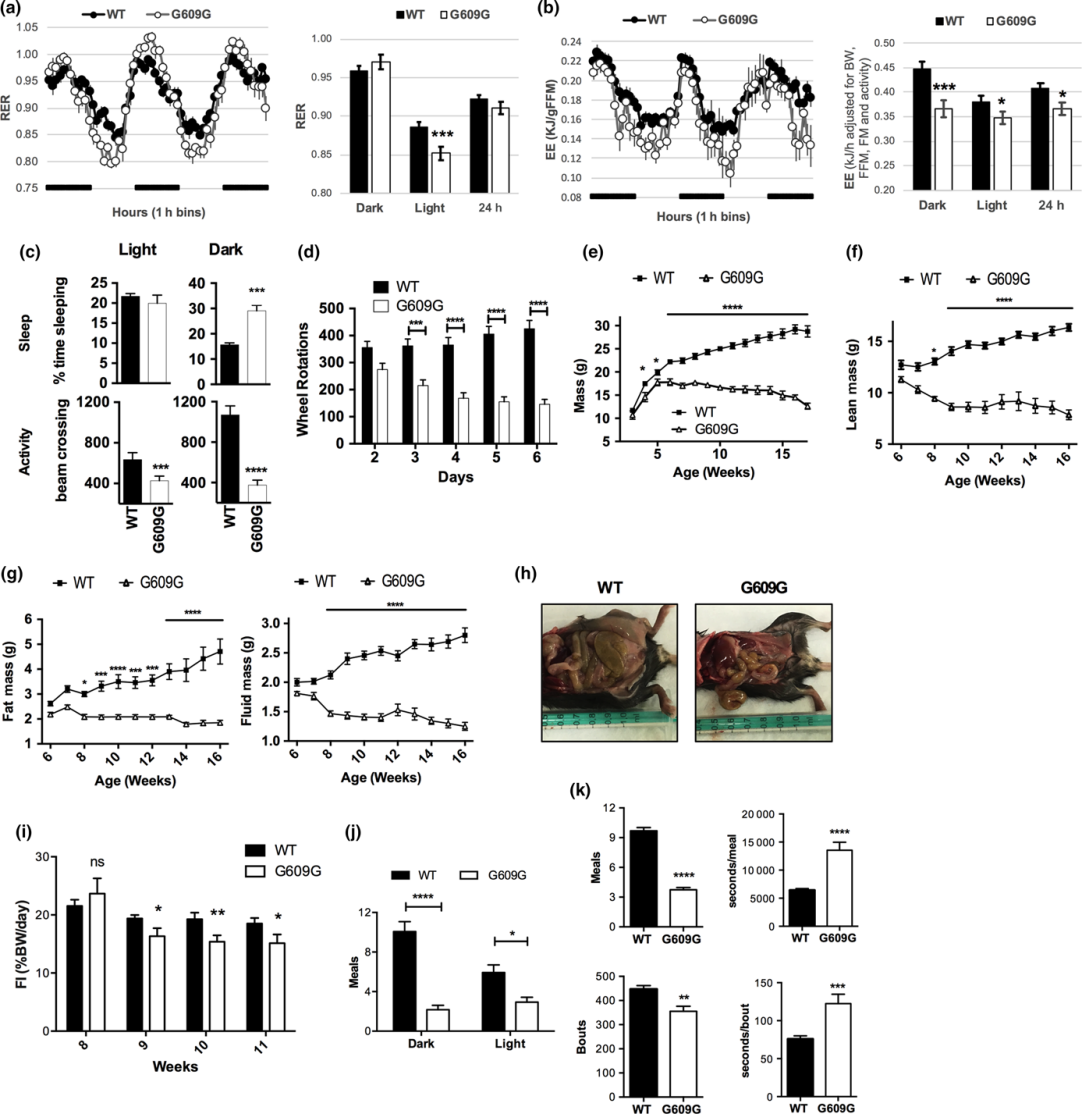
Characterization of metabolism of G609G mice. Comprehensive lab animal monitoring system (CLAMS) was utilized to compare whole body fuel selection, indicated by the respiratory exchange ratio (RER), and energy expenditure between WT and G609G mice at 7 weeks of age (all males). (a) RER was measured every hour for a period of 60 hr in 6 WT mice and 5 G609G mice. Left graph shows raw data in 1‐hr bins, with the dark phase indicated by a black bar. Right graph shows averaged values during light and dark phases, and over 24 hr (right panel). Total number of values averaged: 144 for WT and 120 for G609G mice. RER in WT and G609G mice is significantly different during the lights‐on phase (***p < 0.005). (b) Energy expenditure (EE) in the same mice as in (a). EE is significantly lower in G609G mice compared to WT when adjusted for body mass/composition and physical activity. Left graph shows raw data adjusted for lean mass as 1‐hr bins, with the dark phase indicated by a black bar. Right graph shows the results from an EE analysis using ANCOVA where body weight, body composition –FM (fat mass), FFM (fat‐free mass)‐ and physical activity were included as covariates. An analysis using linear regression indicates body weight, FFM and activity are significant predictors in the model for EE over 24 hr. Total number of values averaged: 144 for WT and 120 for G609G mice. (c) Monitoring of activity and sleep in the same mice as in (a) during the light and dark cycles. (d) Locomotor activity was recorded daily using 7‐week old mice housed in cages with free access to running wheels. *N* = 11 mice per group. (e) Graph shows mass recorded daily for male mice. G609G mice have reduced mass, with a slower rate of gain and lower plateau than WT mice. *N* = 12 mice per group. (f,g) Body mass composition of male WT and G609G mice from 6 to 16 weeks of age. *N* = 10 for each group. (h) Necropsy pictures of 11 weeks‐old male mice. Notice the reduction in abdominal/subcutaneous fat in G609G mice. (i) Food intake (% body weight ingested per day per mouse) calculated daily in single‐housed WT (*N* = 10) and G609G (*N* = 9) mice fed RC diet since weaning. Measurements performed from weeks 8 to 11 of age. (j) Eating behavior as number of meals in the dark and light cycles of WT (*n* = 14) and G609G (*n* = 7) male mice. Recording was performed during 5 days using BiodaQ 2.3 system. (k) Monitoring of total number of meals and bouts (left graphs), and time (seconds) spend per meal/bout over the 5‐days period. In all figures, * denotes *p* value of statistical significance (*p < 0.05; **p < 0.01; ***p < 0.005; ****p < 0.0001), and error bars represent standard error of the mean (*SEM*)

To determine whether progeria mice exhibit metabolic alterations, we utilized a comprehensive lab animal monitoring system (CLAMS) coupled to open circuit calorimetry to assess energy expenditure and whole‐body fuel selection, and NMR to assess body composition. We placed regular chow (RC)‐fed WT and G609G mice in metabolic cages at 7 weeks of age, before the most severe effects of progerin were apparent. The respiratory exchange ratio (RER, the ratio of CO_2_ produced from metabolism and O_2_ intake) reflects the source of energy being oxidized for fuel. During the active (dark) cycle both WT and G609G mice preferentially utilize carbohydrates (RER ~ 1), reflecting the high‐carbohydrate content of the rodent chow diet (58% kJ/carbohydrates). During the resting (light) cycle, G609G mice displayed a more robust decline in RER compared to WT mice, suggesting a stronger preference for utilizing fat or protein as fuel (Figure 1a). The same G609G mice also expended less energy (produced less heat) compared to WT mice (Figure 1b). Energy expenditure (EE) is significantly lower in G609G mice when adjusted for body mass/composition and physical activity, both in the dark and light cycles, and over a 24‐hr period. These differences in RER and EE were accompanied by changes in activity and sleep. G609G mice slept more during the dark cycle and were less active than WT mice at all times (Figure 1c). Importantly, 7‐week old G609G mice behaved differently from WT during voluntary physical exertion (Figure 1d). During the first week after placing mice in wheel running cages, WT mice ran more as they became accustomed to the wheel. In contrast, G609G mice ran less with each consecutive day, indicating potential exhaustion. Our data show that RC‐fed G609G mice have alterations in metabolic rate evidenced by changes in energy expenditure and declining activity, which is consistent with a physiological alteration to conserve energy.

At birth, G609G mice are indistinguishable from their WT littermates, but by 4 weeks of age they are significantly smaller (Figure 1e). This is similar to HGPS patients, who are normal at birth but gradually develop growth defects, often never reaching a total body mass of 30 kg (Kieran, Gordon, & Kleinman, 2007). Finding the source of body mass loss with age in G609G mice is important for understanding its underlying cause. Hence, we monitored body composition in WT and G609G mice weekly using NMR. Interestingly, changes in G609G mass were predominantly driven by loss of lean mass (Figure 1f). By six weeks of age, G609G mice reached their maximum lean and total body mass, and both values progressively declined thereafter. Fat and fluid masses also peaked at six to seven weeks before declining but were relatively smaller contributors to the total body mass loss (Figure 1g). At necropsy, G609G mice had less abdominal and subcutaneous fat than their WT littermates (Figure 1h). This is consistent with previous reports of lipoatrophy in G609G mice due to persistent DNA damage and senescence of adipocytes (Osorio et al., 2011; Revêchon et al., 2017). In summary, our results suggest an energy deficit in G609G mice and features of starvation and cachexia, with mice progressively losing muscle and adipose tissue with age.

To determine if there were differences in food consumption that could explain the decline in body mass over time in G609G mice, we monitored daily food intake (FI = % body weight consumed per day per mouse) of single‐housed mice for 4 weeks. We observed a FI reduction in G609G mice compared to WT by 9 weeks of age (Figure 1i), as well as changes in eating behavior. Normally, mice eat more during the dark cycle, when they are more active, than during the light cycle. In contrast, G609G mice eat at a comparable rate during the dark and light cycles (Figure 1j). Moreover, while the number of meals/bouts are less in G609G mice compared to WT, the time spent in each meal/bout is larger in G609G mice (Figure 1k). In fact, they seemed to be eating constantly while awake, even during the light cycle (Supporting information Video S1). These alterations in FI and eating behavior might contribute to the starvation phenotype of G609G mice fed a RC diet.

### 2.2 HFD ameliorates progerin‐driven mortality

**Figure 2:**
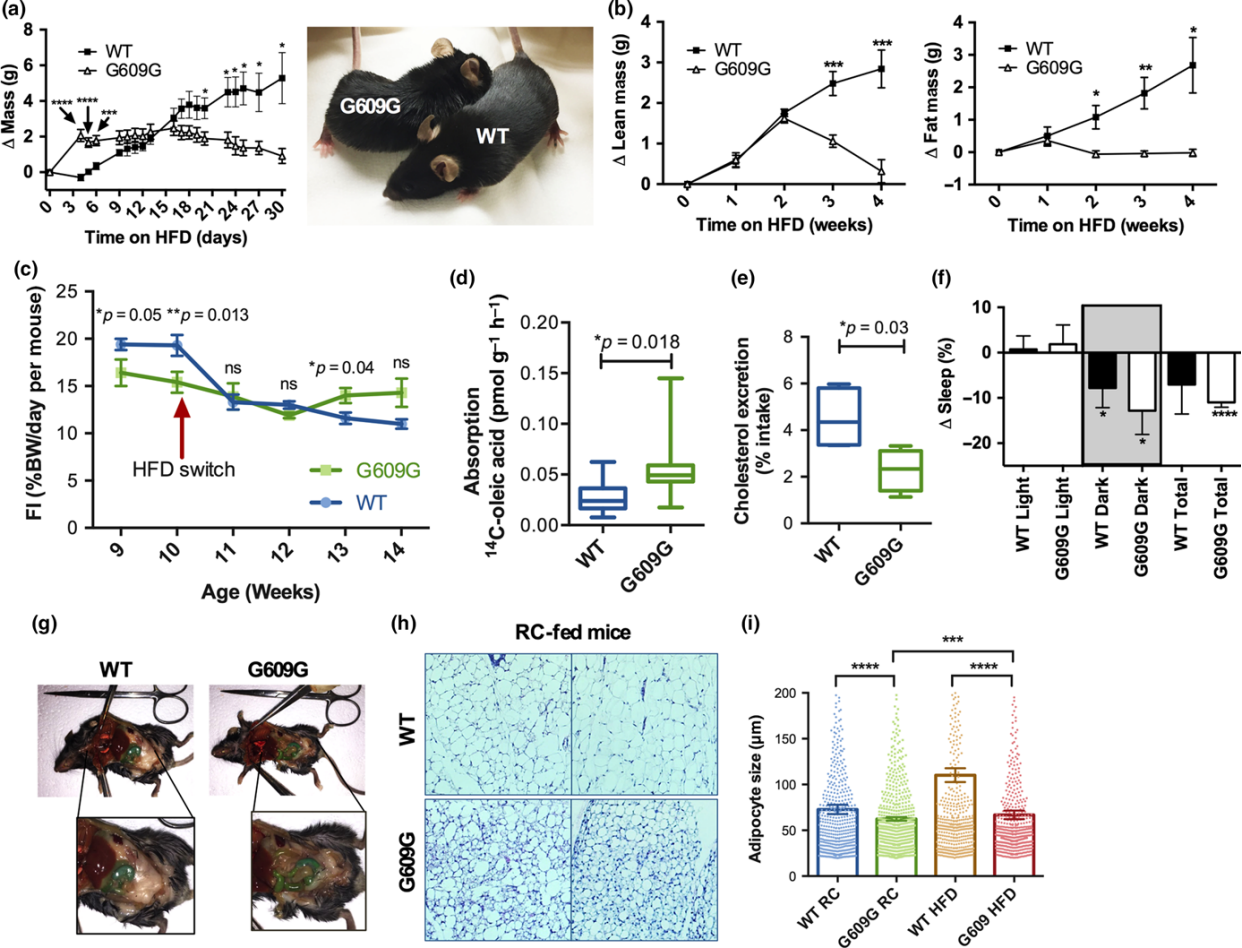
Robust response of progeria mice to high‐fat diet. (a) Male G609G mice (*N* = 5) and WT (*N* = 6) controls were switched to a HFD (60% calories from fat) at 11 weeks of age, and cumulative mass change from starting mass was recorded daily. Picture shows mice 4 days after the switch to HFD, a point at which G609G mice experienced a 15% increase of their body weight. (b) The same mice as in (a), switched to HFD, were placed in NMR weekly to determine lean mass and fat mass changes from the initial mass. (c) Food intake (% body weight ingested per day per mouse) in G609G and WT mice fed RC (weeks 9 and 10) and switched to HFD for 4 weeks (weeks 11–14). Mice were single‐housed, and food consumption monitored daily. Graph shows FI weekly average ± *SEM* (*N* = 5 male mice per group). (d) G609G mice (*N* = 10) and WT controls (*N* = 9) seven weeks of age were administered ^14^C‐oleic acid via gavage and 3 hr later radioactivity in plasma measured. Aborption calculated as pmol of ^14^C‐oleic acid per gram (body weight) per hour. Half mice were males and half females. (e) Single‐housed male G609G mice (*N* = 5) and WT controls (*N* = 5) were switched to a high cholesterol diet (HCD) at 7 weeks of age. Daily feces were collected twenty days after the switch. Note how the amount of cholesterol excreted in the feces is significantly reduced in G609G mice with respect to WT. (f) Change in percentage sleep immediately after mice were switched to HFD compared to sleep pattern on RC. (g) Necropsy images of WT and G609G mice switched from RC to HFD and maintained on HFD for four weeks. Notice adipose tissue is evident in abdominal cavity of G609G mice, which was not previously present for mice on RC (Figure 1d). (h) Images from histology of H&E stained adipose tissue from WT and G609G mice fed RC. (i) Graph shows quantification of adipocytes size in G609G (*N* = 9) and WT (*N* = 10) mice fed RC up to 11 weeks of age, and in mice switched from RC to HFD at 11 weeks of age and maintained in HFD for 4 weeks (*N* = 5 in each group of both G609G and WT mice)

G609G mice exhibit features of both starvation, which stems from lack of adequate energy intake, and cachexia, driven by an intrinsic process independent of energy intake (Argiles, Busquets, Stemmler, & Lopez‐Soriano, 2014; Thomas, 2007). Since starvation can be ameliorated by increased energy consumption, while cachexia generally cannot, we tested whether a high‐fat diet (HFD; 60% calorie from fat) could improve the G609G mouse phenotype. Hence, as G609G mice approached terminal stage (11 weeks of age), we switched their diet from RC to HFD. Remarkably, the G609G mice immediately responded in a positive manner. In 4 days, the G609G mice gained two grams, a 15% increase in total body mass, while the WT mice showed no change in body mass (Figure 2a). G609G mice fed HFD maintained body mass for three weeks but began to fall by four weeks. NMR analysis indicated that increased lean mass was primarily responsible for increased body mass in G609G animals (Figure 2b). Fat mass was maintained in G609G mice while on HFD. This is in contrast with the WT mice, which gained fat mass at a linear rate over the 4‐week HFD regimen. Importantly, while in this particular experiment, all G609G mice on RC were dead before reaching 100 days of age, all mice on HFD were alive and seemed healthy 30 days after the HFD switch.

We then determined if there were differences in daily FI that could explain the improvement after the switch to HFD. Figure 2c represents weekly averages of daily FI. WT mice switched to HFD exhibit a marked reduction in FI, given the higher caloric content of the HFD. In contrast, G609G mice continue to ingest similar amounts of food after the switch, and with the greater caloric density of the HFD they are consuming increased calories (Figure 2c). Thus, ingesting HFD, with higher caloric content, is able to improve morbidity and rescue early lethality, suggesting that starvation is contributing to the decline and early death of G609G mice. In addition to changes in FI, we tested for deficiencies in nutrient absorption in G609G mice with respect to WT mice, as intestinal malabsorption could contribute to starvation and cachexia phenotypes. WT and G609G mice were administered a radioactive lipid (^14^C‐oleic acid) by oral gavage, followed by detection of radioactivity in plasma. Interestingly, G609G mice absorb significantly more fatty acids than WT mice (Figure 2d). We also monitored cholesterol excretion in feces. WT and G609G mice were switched to a high‐cholesterol diet, and the amount of cholesterol excreted in the feces in 1 day quantitated 20 days after the switch. G609G mice displayed less excretion of cholesterol compared to WT mice (Figure 2e), suggesting a potential increase in absorption. We propose that increased nutrient absorption by G609G mice might be a mechanism to compensate for reduced FI in these mice.

Importantly, HFD feeding resulted in additional phenotypic improvements in G609G mice: they slept less and were less lethargic after switching to HFD than before starting the diet (Figure 2f and Supporting information Video S2). At necropsy, HFD‐fed G609G animals exhibited abdominal fat which was not evident in RC‐fed mice, although fat deposits were still reduced compared to either RC‐ or HFD‐fed WT mice (Figure 1c and 2g). Adipose tissue histology revealed decreased adipocyte size in RC‐fed G609G mice with respect to WT mice (Figure 2h). Mice switched to HFD show an increase in adipocyte size, however the increase is more robust in WT mice (Figure 2i). These results are consistent with severe lipoatrophy in progeria mice, which is ameliorated by HFD. Overall, our data suggest that G609G mouse mortality is driven by starvation and cachexia. While switching G609G mice from RC to HFD provides the source of energy needed to extend their lifespan, the beneficial effect appears to be only temporary.

### 2.3 HFD doubles lifespan of progeria mice

**Figure 3:**
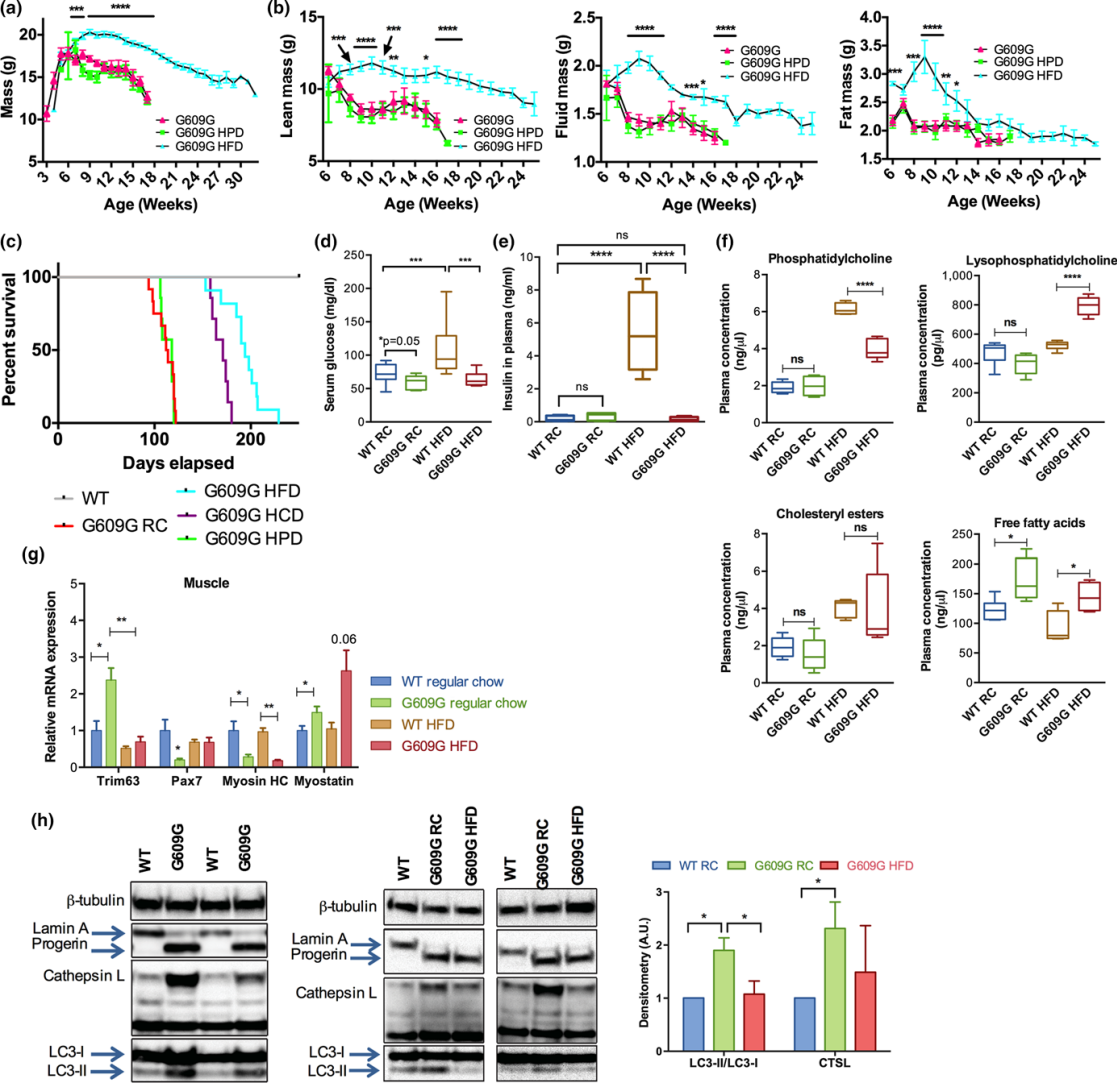
Delayed aging phenotypes and extended lifespan of G609G mice fed HFD. (a) Mass was recorded daily for male G609G mice fed RC (*N* = 12), HFD (*N* = 11), or high‐protein diet (HPD; *N* = 7) since weaning. Note the sustained increase in body mass of G609G mice on HFD. (b) Lean, fluid, and fat mass composition as measured by NMR in male G609G mice fed RC, HFD or HPD from 6 to 25 weeks of age. (c) Kaplan–Meier survival curves of male WT and G609G mice fed RC (*N* = 12), HFD (*N* = 11), HPD (*N* = 7), and HCD (*N* = 7; a diet consisting of 40% fat content + cholesterol). (d) Blood glucose monitored for groups of mice fed RC or HFD since weaning and fasted for 6 hours: WT RC (*N* = 13), G609G RC (*N* = 12), WT HFD (*N* = 7), and G609G HFD (*N* = 10). Analysis at 11 weeks of age. (e) Insulin levels measured for WT and G609G mice fed RC (~11 weeks of age) or HFD (~22 weeks of age): WT RC (*N* = 9), G609G RC (*N* = 7), WT HFD (*N* = 8), G609G HFD (*N* = 4). Mice were fasted for 3 hours prior to collecting blood for analyses. WT mice develop hyperglycemia and hyperinsulinemia on HFD, while G609G mice do not. (f) Concentrations of phosphatidylcholines, lysophosphatidylcholines, cholesteryl esters, and free fatty acids as determined by mass spectrometry. Female G609G and WT mice were used for this study. Mice were either fed RC since weaning or switched from RC to HFD at 11 weeks of age for four weeks (as in Figure 2a). Samples were collected at 11 weeks of age in mice on RC and at 14 weeks of age in mice switched to HFD. *N* = 5 in each group. (g) qRT‐PCR analysis on muscle samples (quadriceps) from WT (*N* = 6) or G609G (*N* = 4) mice fed RC (11 weeks of age) or HFD (22 weeks of age). (h) Immunoblotting on proteins isolated from WT or G609G mouse quadriceps at necropsy. Notice expression of progerin and increased expression of CTSL and LC3‐II in G609G mice (left panel), which are ameliorated on HFD (right panels). Graph shows densitometry of immunoblots (average ± *SEM*) performed in WT (*n* = 6) and G609G (*N* = 6) mice fed RC (11 weeks old) and G609G fed HFD (*N* = 4 and 22 weeks old) mice. Note the increase in LC3‐II/LC3‐I ratio and CTSL levels in G609G mice fed RC, and the decrease in G609G mice fed HFD

Given the benefits of switching RC‐fed G609G mice nearing death to HFD, we tested whether early HFD feeding had a more robust and sustained effect, and whether other diets might affect morbidity and mortality. To this end, we allocated mice to one of the three groups after weaning: RC, HFD, or a high‐protein diet (HPD). To ensure that phenotypic differences that developed between diets were due to nutritional differences and not edibility and palatability, all diets were provided both as pellets and grounded, so mice across all diets had access to soft food. Tracking of body mass (Figure 3a) revealed that mice in the HPD and RC groups behaved similarly, reaching an average maximum mass of 18.2 and 17.8 g, respectively, at 6 weeks of age and declining thereafter. In contrast, HFD‐fed G609G animals reached a maximum body mass of 20.3 g, 14% higher than that of RC‐fed mice, at 9 weeks. Thus, HFD raised the maximum body weight and delayed the body mass inflection point, indicating that HFD slows the decline of G609G mice. Lean, fluid, and fat masses followed similar trends (Figure 3b). RC‐fed and HFD‐fed mice reached similar maximum lean masses, but with a difference of 4 weeks (at 6 and 10 weeks, respectively), demonstrating that HFD delays lean mass loss in G609G mice. Maximum fluid mass was greater in HFD‐fed mice and was reached at week nine compared to week six in RC and HPD mice. Fat mass mirrored this pattern as well.

The most striking effect of the different diets was on lifespan (Figure 3c). G609G mice maintained on RC and HPD lived 111 and 113 days on average respectively. Their maximum lifespans were 122 and 120 days respectively. However, as the RC and HPD mice began to die, HFD mice remained in robust health. In fact, G609G mice continued to thrive and lived for 193 days on average (~75% increase in lifespan). Notably, this is the largest lifespan extension reported to date in G609G mice. The maximum lifespan was 229 days, which doubled the average lifespan of RC‐fed mice. Given this robust effect of HFD, we tested whether a high‐cholesterol diet (HCD) might impact lifespan, since this diet has an intermediate calorie and fat content compared to HFD. Interestingly, mice on HCD lived an average of 169 days, which was significantly longer than the HPD and RC, but still less than the HFD. Therefore, diets with increased energy content had the greatest beneficial effect on G609G mice. Fat content was also important, as lifespan was identical for G609G mice in the fat‐equivalent HPD and RC, despite the HPD providing 30% more energy content overall. We conclude that increasing dietary energy content, and especially fat content, delays the onset of starvation and cachexia driving RC‐fed G609G mouse death, thus significantly extending their lifespan.

### 2.4 HFD induces metabolic changes in G609G mice

Normally, increased calorie consumption leads to a slew of detrimental side effects, including hyperglycemia and hyperinsulinemia. We tested whether HFD consumption in G609G mice elicits detrimental metabolic consequences normally associated with these diets. While WT mice became hyperglycemic on HFD, G609G mice at 11 weeks of age treated for 2 months with HFD maintained relatively normal levels of blood glucose (Figure 3d). Moreover, G609G mice on RC exhibit lower insulin levels than WT mice. Importantly, the robust increase in insulin levels in WT mice fed HFD is not observed in G609G mice (Figure 3e). G609G mice on RC also displayed impaired glucose tolerance that was normalized by the HFD and the G609G mice on HFD did not display signs of insulin resistance typically observed in WT mice on this diet (Supporting information Figure S1). A pyruvate tolerance test revealed a lower capacity of gluconeogenesis in G609G mice relative to WT mice that was not significantly altered by the diet. These data indicate a distinct metabolic response of G609G mice to HFD compared to WT mice.

To further investigate the impact of HFD on G609G mice, we performed complete lipidomics using mass spectrometry on plasma isolated from WT and G609G mice on RC and HFD (Figure 3f and Supporting information Figure S2). Although the differences between the WT and G609G mouse lipids levels were few, there were several key differences observed. Phosphatidylcholine (PC), an abundant component of lipid bilayers and lipoproteins, was elevated in both WT and G609G mice upon HFD feeding, but the increase was significantly lower in G609G mice. Lysophosphatidylcholine (LPC), a metabolite product of PC, was selectively elevated in only the HFD‐fed G609G mice suggesting an increase in PC degradation. Cholesteryl esters increased in a similar fashion in both WT and G609G mice when fed HFD. Interestingly, free fatty acid (FFA) species were increased in G609G mice relative to WT mice, both on RC and HFD. FFAs are known to increase with starvation (Yaffee, Gold, & Sampugna, 1980). The increase in FFAs in G609G mice could also reflect lipolysis of adipose tissue.

Muscle breakdown and dysfunction is a key characteristic of cachexia (Thomas, 2007). Since we observed cachectic tendencies in G609G mice, we monitored pathways with key roles in muscle homeostasis. We found that expression of genes involved in muscle catabolism and structure were altered in G609G mice relative to WT mice and these alterations were ameliorated by HFD (Figure 3g). *Trim63*, important for muscle protein breakdown and inhibition of protein synthesis, is upregulated in G609G mice on RC, and HFD restores normal levels of *Trim63* expression. Interestingly, this gene is frequently upregulated in cachexia (Cai et al., 2004; Yuan et al., 2015). G609G mice also exhibit downregulation of *Pax7*, a key gene for satellite cell survival (Lepper, Partridge, & Fan, 2011; Sambasivan et al., 2011). Satellite cells are necessary for skeletal muscle regeneration and repair, and *Pax7* loss can cause major disruptions in adult myogenesis (Maltzahn, Jones, Parks, & Rudnicki, 2013). Reduced *Pax7* expression in G609G mice on RC suggests a likely deficiency in muscle repair capacity. Importantly, mice on HFD show a marked increase in *Pax7* expression, reaching levels similar to WT mice. Other changes in G609G muscle gene expression were not affected by HFD. Myosin heavy chain (*Mhc*), the motor protein of muscle thick filaments, is essential for proper muscle function. In G609G mice, this gene is downregulated, and its expression is not increased on HFD. Likewise, myostatin (*Mstn*), a negative regulator of muscle growth and development that is associated with cachexia (Zhou et al., 2010), is increased in G609G mice, and HFD does not reduce its levels. Therefore, while HFD is able to improve some gene expression alterations in muscle, others remain unchanged.

We also found protein alterations consistent with muscle disease in G609G mice (Figure 3h). Cathepsin L (*Ctsl*) is a lysosomal cysteine protease involved in protein degradation, known to be upregulated during muscle wasting (Deval et al., 2001). Interestingly, cathepsin L protein levels are increased in G609G mice, and reduced by HFD. Similarly, LC3, an important marker of autophagy, is increased in G609G mice on RC. This could reflect activation of autophagy in order to generate energy. G609G mice on the HFD exhibit reduced LC3 expression, suggesting that these mice are obtaining sufficient energy from the diet. These analyses indicate that G609G mice phenotype is characterized by features of both cachexia and starvation.

### 2.5 HFD‐fed G609G mice develop a more severe progeroid phenotype

**Figure 4:**
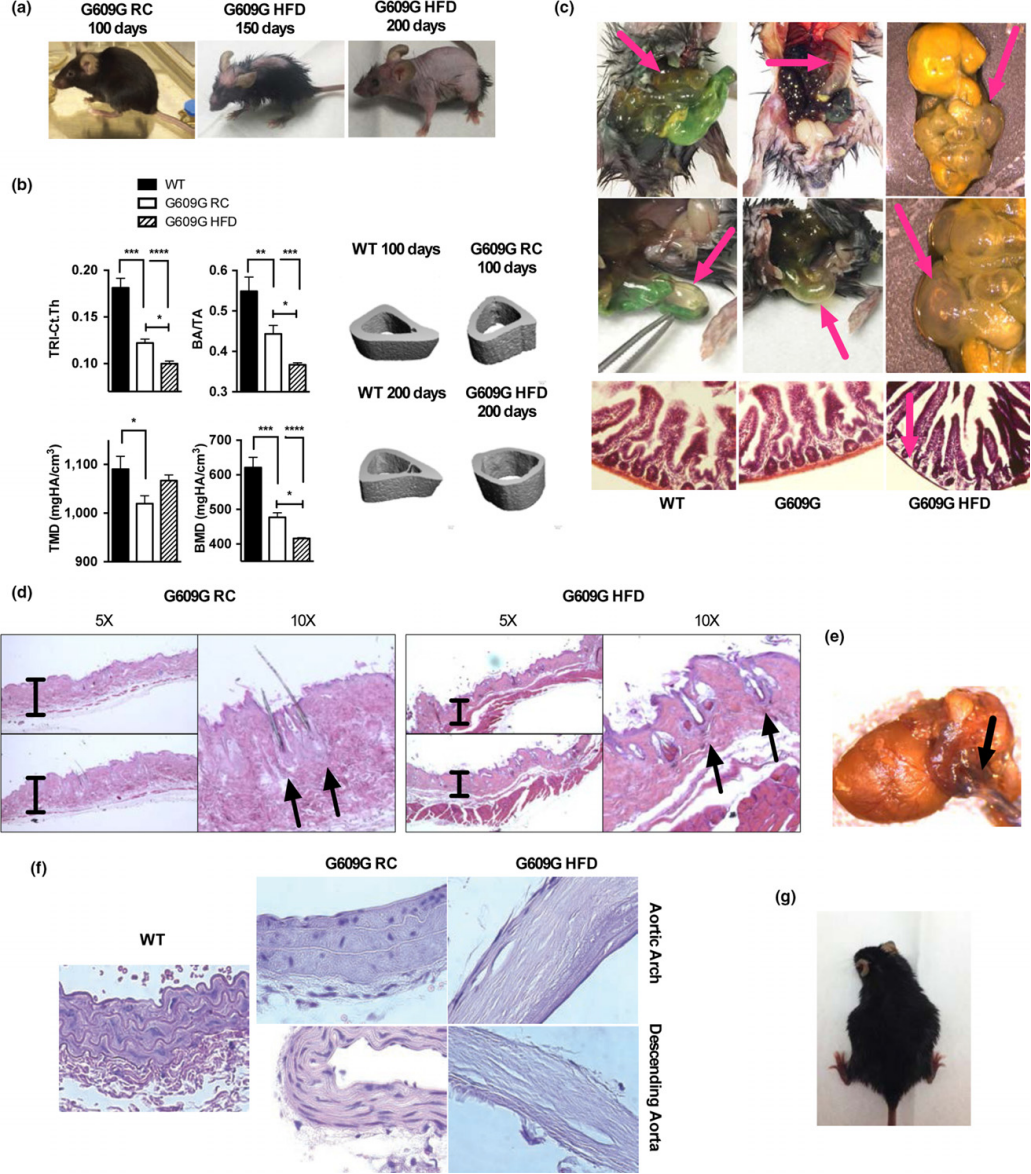
HFD‐fed G609G mice embody characteristics of human phenotype. (a) Pictures were taken of G609G mice on RC near death (100 days), and on HFD at 150 and 200 days of age. Note the progressive development of progeroid features, especially allopecia. (b) X‐ray microCT was conducted on tibiae of WT mice, G609G mice fed RC (100 days of age), and G609G mice fed HFD (200 days of age). Cortical analyses were performed to quantify total area (TA), total bone area (BA), cortical thickness (Ct.Th, TRI method), bone mineral density (BMD), and tissue mineral density (TMD). Pictures are representative of tibiae samples analyzed. (c) Pictures of intestines taken at necropsy of G609G mice on HFD ~200 days old, and histological analysis performed by H&E staining. Note the alterations in intestine morphology, and the loss of muscularis by histology. (d) Histology of skin with H&E staining shows examples of exacerbation of alterations in hair shafts of HFD‐fed G609G mice (~200 days of age) compared to RC‐fed mice (~100 days of age), consistent with robust alopecia. Pictures were taken at two different magnifications (5× and 10×). (e) Picture of heart and aorta taken from a HFD‐fed G609G mouse (~200 days of age) at necropsy. (f) Histology of aortas with H&E staining shows examples of acellular and mineralized aortas in G609G mice on HFD (~200 days of age), compared to RC‐fed WT and G609G mice (~100 days of age). Pictures taken at 40× magnification. (g) Picture shows an example of a pregnant HFD‐fed female G609G mouse (third pregnancy). None of the RC‐fed G609G females became pregnant in our study

While RC‐fed G609G mice recapitulate many aspects of the human disease, including skin, cardiovascular, and bone alterations, the severity of these phenotypes is not as pronounced as in human patients. Interestingly, as HFD‐fed progeria mice aged, we noticed that these mice progressively developed pathologies that mimicked the severity of human disease, such as thinner skin and prominent alopecia, eye defects, and more pronounced bone dysplasia (Figure 4a). Thus, HFD did not prevent the development of progeroid phenotypes; rather, the extended lifespan on HFD unmasked some critical pathological characteristics of human condition.

To characterize these pathological changes more completely and understand the prolonged effect of progerin in vivo, we examined the bone of progeria mice maintained on HFD vs RC by conducting µCT on the whole animal (Supporting information Figure S3) and in tibias (Figure 4b). Interestingly, most tibia bone parameters continued to degenerate with increasing age of G609G mice on HFD. HFD‐fed G609G mice at 200 days of age had decreased cortical thickness (Ct. Th), bone area to total area (BA/TA), and bone mineral density (BMD) compared to both WT and RC‐fed G609G mice at 100 days of age, indicating progressive thinning of bone.

Another organ with noticeable degeneration at necropsy was the intestine (Figure 4c), which appeared severely inflated and filled with air in HFD‐fed G609G mice of ~200 days of age. These intestines had thinner muscularis (by histology) and, at certain areas, were almost transparent. In some, it also appeared that the mice had a severely swollen appendix. In every animal examined, there were obvious intestinal alterations and defects.

G609G mice on HFD also developed pronounced skin alterations, with progressive and severe alopecia (Figure 4a). Histological analysis of skin samples revealed significantly diminished subcutaneous fat and thinner skin, which was obvious in RC‐fed mice, but accentuated in HFD‐fed mice as they aged (Figure 4d). HFD‐fed G609G mice also showed fewer hair follicles and hair shafts in the follicles, and many follicles appeared to have disordered differentiation. In addition, there were cystic growths in the dermis, suggesting atrophy of hair follicles in HFD‐fed G609G mice.

Previous studies in G609G mice fed a normal diet showed cardiovascular alterations, including calcification of the aorta and reduced number of VSMCs in the aortic branch (Osorio et al., 2011; Villa‐Bellosta et al., 2013). We find that HFD‐fed G609G mice developed aortic defects with a severity not observed previously in any mouse model of progeria. At necropsy, the aortas appear significantly stiffer in HFD‐fed G609G mice (~200 days of age) than in RC‐fed mice (~100 days of age). Some of the HFD‐fed mice showed gross morphological abnormalities, including in some situations where the aorta became fused to the heart (Figure 4e). However, the most dramatic phenotype was observed in histological sections (Figure 4f and Supporting information Figure S4). The ascending aorta, descending aorta, and aortic arch showed mineralization of the media, thickening of the adventitia, and broad acellularity in both the media and the adventitia, with a profound loss of VSMCs. In addition, elastic fibers appeared stretched, a phenotype consistent with alterations in aortic mechanical properties. This severe aortic phenotype could play a major role in the decline and death of G609G mice on HFD. Of note, despite the aortic stiffening, we did not find signs of atherosclerosis in the aorta, but this was not unexpected given that the mice were not in a hypercholesterolemic background.

Another interesting observation was that, unlike the G609G mice maintained on RC, HFD‐fed G609G female mice were able to become pregnant and deliver viable litters (Figure 4g). G609G mice have been previously characterized as infertile and our observation is consistent with an improvement in their health due to maintenance on high energy diet. While these mothers have been unable to care for their litters to this point, possibly as a result of lack of milk production, the ability of these females to carry a full litter to term is impressive. In summary, HFD improves the G609G mouse phenotype in many ways. Most significant is the fact that G609G HFD‐fed mice overcome the starvation phenotype driving early lethality to unveil important human‐like phenotypes.

## 3 DISCUSSION

Our study demonstrates the importance of caloric intake to the development of various phenotypes in G609G mice. The finding that HFD results in the largest extension of life recorded to date in an HGPS mouse model suggests that a nutritional/nutraceutical approach could provide a powerful tool for modulating disease course in both mice and humans. Interestingly, the Progeria Research Foundation recommends feeding high calorie foods to HGPS patients:

Children with Progeria actually eat enough calories to grow, but the basic disease process in Progeria does not allow them to grow normally. Some parents also report that the children tend to take in smaller, more frequent meals. Therefore, the goal is to give them nutritious and high calorie foods and supplements.

Our finding that dietary intervention results in both lifespan extension in G609G mice and in the development of aging phenotypes with a robustness that emulates the human syndrome is in line with studies in other mouse models of progeria. For instance, in mouse models of Cockayne syndrome, feeding pregnant dams HFD rescues death around weaning, thus revealing symptoms of neurodegeneration later in life that are much more emblematic of the human disease (Brace et al., 2013). HFD was also efficacious against symptoms at metabolic, transcriptomic, and behavioral levels in these mice (Scheibye‐Knudsen et al., 2014). Altogether, these studies suggest that mouse models of progeria are especially susceptible to food/caloric intake, and that ensuing proper energy intake can allow in some cases a better recapitulation of the pathologies of the human diseases.

Our study also shows that HFD alone cannot override the long‐term detrimental effects of progerin expression in tissues, and that the effect of other strategies with known benefits reducing progerin toxicity in mice should be tested in combination with HFD. One of the first strategies with reported benefits in progeria mice was the treatment with farnesyltransferase inhibitors (FTIs). The success in mice led to the use of lonafarnib in the first clinical trial in HGPS patients. Lonafarnib treatment resulted in a modest improvement of mean survival (1.6 years) and is currently the standard of care for HGPS patients (Capell et al., 2008; Fong et al., 2006; Gordon et al., 2012, 2014). Subsequent studies have identified different strategies with benefit in progeria mice, including sodium salicylate (Osorio et al., 2012), splicing therapies (Osorio et al., 2011), reprogramming (Ocampo et al., 2016), and reduced NF‐κB signaling (Osorio et al., 2012). Remarkably, the reported effects of all these strategies ameliorating disease and increasing lifespan are far more modest than the effects of HFD. This suggests that these strategies might not be able to ameliorate the combined features of cachexia and starvation in the progeria mice as well as HFD. Thus, the benefit of these strategies, and new strategies being developed to counteract progerin toxicity in vivo (rapamycin, antioxidants, remodelin, all‐trans retinoic acid, 1,25α‐dihydroxy‐vitamin D_3_, pyrophosphate, etc; Cao et al., 2011; Gabriel, Roedl, Gordon, & Djabali, 2015; Kreienkamp et al., 2016, 2018; Larrieu, Britton, Demir, Rodriguez, & Jackson, 2014; Pellegrini et al., 2015; Villa‐Bellosta et al., 2013), should be investigated in combination with HFD.

Overall, our findings suggest that metabolic features in HGPS patients are an important area for investigation, as they might have a more important contribution to disease pathology than previously thought. For instance, further studies are needed to identify molecular mechanisms responsible for the starvation/cachexia‐like phenotypes in progeria mice. In addition, our study identified genes involved in tissue homeostasis that are dysregulated in G609G mice. Musculoskeletal problems remain a major challenge for HGPS patients. Therefore, identifying transcriptome and proteome alterations that might be targeted pharmacologically or nutraceutically would be extremely useful for HGPS patients. In human disease, stem cell depletion is considered a key contributor to HGPS phenotype (Rosengardten, McKenna, Grochova, & Eriksson, 2011). There is growing evidence that energy balance and metabolic status are critical for stem cell function and life‐long tissue renewal (Suda, Takubo, & Semenza, 2011). Future studies need to address whether metabolic alterations drive stem cell exhaustion in progeria mice, and whether HFD improves stem cell function, allowing slower aging of these mice. Moreover, our lipidomics studies, which represent the first completed in vivo lipidomic studies in progeria, reveal that lipid alterations, if replicated in humans, might be a substantial contributor to atherosclerosis in HGPS patients. Particularly since LPCs are elevated on HFD, which resembles features of the typical western‐diet, greater attention should be paid to lipids and LPCs in HGPS phenotype, especially given the known contribution of LPCs to atherosclerosis (Schmitz & Ruebsaamen, 2010).

In summary, we propose that the combination of nutraceutical and pharmacological strategies might provide the most benefit for HGPS treatment, by targeting the multifactorial detrimental effects of progerin expression in vivo.

## 4 MATERIALS AND METHODS

### 4.1 Mouse model of progeria

Mice carrying the human HGPS mutation (G609G) were generated in the laboratory of Carlos Lopez‐Otin (C57BL/6 strain). Heterozygous *Lmna^G609G/+^* female and male mice were crossed to obtain WT mice and mice carrying the mutation in homozygosis. *Lmna^G609G/G609G^* (herein G609G mice) are the focus of this study. Littermates of the same sex were randomly assigned to experimental groups.

G609G mice gain body mass at a slower rate than age‐matched WT mice and are significantly smaller by four weeks of age. G609G mice are infertile and show abnormal posture and marked curvature of the spine, dying at ~100 days when fed a RC diet. Analyses of phenotypes of mice were performed at different times throughout their lifespan, and at time of death. Procedures performed in G609G mice include: blood collection, food and water modifications, food deprivation, NMR, performance tests, single housing, and tissue collection.

All animal studies were approved (protocol #2299) and conducted in accordance with the Animal Studies Committee at Saint Louis University (Dr. John C. Chrivia, Ph.D. is the Institutional Animal Care and Use Committee Chair). The mice used in these studies were housed at a constant temperature of 23°C with food and water provided ad libitum under a 12:12 light‐dark cycle, unless otherwise specified. Food intake and body weight were monitored daily in these experiments and body composition was measured every week by NMR (Bruker BioSpinLF50). Animals were fed either regular chow, high‐fat diet, high‐cholesterol diet, or high‐protein diet. The composition of the different diets, as well as the catalog numbers of each diet for more in‐depth information, is shown in the following table:

**Table:** 

	Regular chow (RC)	High protein diet (HPD)	High cholesterol diet (HCD)	High fat diet (HFD)
Supplier	LabDiet	ResearchDiets	Tektad	ResearchDiets
Diet number	5,053	D17010902	TD.05305	D12492
kcal/g	3.0	3.9	4.5	5.2
%kcal protein	24.5	60.0	15.2	20.0
%kcal carbohydrates	62.4	30.0	42.7	20.0
%kcal fat	13.1	10.0	42.0	60.0

### 4.2 Comprehensive lab animal monitoring system (CLAMS)

The Columbus Instruments (Columbus, OH) CLAMS system was utilized to monitor the metabolic parameters of mice (male or female G609G and WT littermates, at 7 weeks for chow or HFD). Five G609G mice and six age‐matched WT mice were housed individually in metabolic cages on a 12 hr day–night cycle, fed with either regular chow (RC) or high fat diet (HFD). Mice were acclimated for 5 days in the CLAMS unit. The hourly or average values during light and dark period were calculated. Two‐way ANOVA follow by Bonferroni post‐test was used to calculate the p value of statistical significance.

### 4.3 Assessment of locomotor activity

Locomotor activity was assessed using mice housed in cages with free access to running wheels. Briefly, after a 2 days’ acclimation period to wheel‐equipped cages in a 12:12 light‐dark (LD), locomotor activity was recorded over a 1‐week period. Wheel running data were analyzed using Clocklab software (Actimetrics, Evanston, IL).

### 4.4 Assessment of food intake and meal pattern analysis

Meal structure was examined using an automated system for continuous monitoring of food consumption (BiodaQ 2.3; Research Diets Inc., New Brunswick, NJ) and BiodaQ 2.3 software. “Bouts” indicate disturbance of the hopper and instability in scale readings suggesting approach and investigation; actual changes in food mass were used to estimate meal size. Meals were defined as bouts occurring within 5 min of each other and consumption of ≥0.02 g. After 2 days of acclimation in BiodaQ cages, feeding behavior was established using 5 days of recordings.

### 4.5 Fecal analysis

For the analysis of fecal lipids, feces were collected from mice housed individually in metabolic cages over a 24‐hr period. One‐hundred‐milligram aliquots of feces were cleaned and dried for 1 hr at 70°C, incubated with 2 ml of chloroform‐methanol (2:1) for 30 min at 60°C with constant agitation, and then centrifuged. Water (1 ml) was added to the supernatant, and following vortexing, phase separation was induced by low‐speed centrifugation (2,000 rpm for 10 min). The lower chloroform phase was then removed and transferred to a new tube, and the sample was evaporated to dryness. Samples were then resuspended in 500 μl chloroform‐1% Triton X‐100, evaporated to dryness, and finally resuspended in 500 μl of water, so that the final solvent was 1% Triton X‐100 in water.

### 4.6 Intestinal absorption of radioactive lipids

WT and G609G mice fed RC diet were fasted overnight, then gavaged with 100 µl olive oil containing 1 mCi/ml [^14^C]‐oleic acid. Blood was collected from the inferior vena cava exactly 3 hr post gavage, and radioactivity counted by liquid scintillation and normalized to body weight.

### 4.7 Glucose, Insulin, and Pyruvate tolerance tests

After 6‐hr fast, WT and G609G mice (*n* = 8) were injected intraperitoneally with glucose (2 g/kg of fat free mass), insulin (0.75 U/kg of fat free mass), or pyruvate (2 g/kg of fat free mass; Sigma‐Aldrich, St Louis MO, USA) to perform glucose tolerance test (GTT), insulin tolerance test (ITT) or pyruvate tolerance test (PTT) respectively. Blood was collected by tail snip and glucose was measure before the injection (*t* = 0 min) and 15, 30, 60, and 120 min after injection using OneTouch Ultra®2 glucometer.

### 4.8 Histology

The proximal thoracic aorta was excissed, cleaned from surrounding fat tissue, and divided into three fragments: ascending (from heart to brachiocephalic braching), arch (from brachiocephalic to left subclavian braching), and descending (after left subclavian braching). These three segments were fixed 5 days in buffer containing 10% formalin, 5% sucrose, and 2 mmol/L EDTA, and subsequently embedded in paraffin blocks. Four‐micrometer sections were then stained with hematoxylin and eosin, following standard techniques.

### 4.9 Immunoblotting

Mouse quadriceps were extracted at necropsy and flash frozen. Quadriceps were then thawed and homogenized in 8 M urea. 80 mg of total protein was loaded on 4%–15% Criterion TGX Gel. List of antibodies used for immunoblotting in Supporting information Reagents Table S1.

### 4.10 Quantitative reverse‐transcription PCR

RNA was reverse transcribed to make cDNA using qScript™ cDNA Synthesis Kit (Quanta biosciences) according to the manufacturer's instructions. Real‐time PCR was performed using a SYBR‐green PCR master mix kit (SYBR SELECT MASTER MIX; Life Technologies). List of primers used in Supporting information Reagents Table S1.

### 4.11 Statistical analysis

Quantification details of all the experiments performed, including the gender and number of animals analyzed (*N*), are indicated in the figure legends. Statistical analyses were performed using Prism 7 (GraphPad Software, La Jolla, CA) or SPSS Statistics Version 23 (IBM). For simple comparisons between multiple groups, we performed ANOVA; if *p* < 0.05 significance between groups was determined using post hoc analysis. For comparisons between groups, Student's *t* test was used with Bonferroni correction. Data are presented as means ± *SEM*. Survival curves were generated using the Kaplan–Meier method. Power was calculated using https://www.statstodo.com/SSizSurvival Pgm.php with the following data/assumptions: RC *n* = 12, HFD *n* = 11, HPD *n* = 7, HCD *n* = 7, alpha = 0.05. RC vs HFD 2 tailed power = 0.94, RC vs HCD 2 tailed power = 0.94, HFD vs HCD 2 tailed power = 0.94, RC vs HPD tailed power = 0.05. In all figures, * denotes *p* value of statistical significance (*p < 0.05; **p < 0.01; ***p < 0.005; ****p < 0.0001).
