# Supplementary material for: Doubled lifespan and patient‐like pathologies in progeria mice fed high‐fat diet
Source: Aging Cell. 2018 Dec 12;18(1):e12852. doi: 10.1111/acel.12852 (PMC6351834; doi:10.1111/acel.12852)
Supplement: Supplementary file 1 [file ACEL-18-e12852-s001.docx]

**Supplementary Materials**

Supplementary Materials and Methods:

Lipidomics

Micro-computed tomography

Reagents table

Supplementary figures:

Fig. S1. Glucose, insulin, and pyruvate tolerance tests.

Fig. S2. Lipidomic studies in RC- and HFD-fed WT and G609G mice.

Fig S3. X-ray microCT.

Fig S4. Aortas of HFD-fed G609G mice.

Other supplemental files:

Movie S1. Improvement of phenotypes of G609G mice on HFD.

Movie S2. Different feeding behavior between WT and G609G mice fed RC.

Supplementary Materials and Methods:

***Lipidomics.*** Lipids were extracted in the presence of internal standards (*vide infra*) from OMVs, or alternatively using a conventional Bligh Dyer extraction. Fatty acid analysis was performed with gas chromatography-mass spectrometry, and phospholipid analysis was performed with shotgun lipidomics with electrospray ionization mass spectrometry on a triple quadrupole instrument. Lipid class internal standards included eicosanoic acid, 1-0-heptadecanoyl-sn-glycero-3-phosphocholine, 1,2-dieicosanoyl-sn-glycero-3-phosphocholine, and 1,2-ditetradecanoyl-sn-glycero-3-phosphoethanolamine. Fatty acids were converted to their pentafluorobenzyl esters and then were subsequently quantified using GC-MS with negative ion chemical ionization with methane as the reactant gas. For phospholipids, lipid extracts were diluted in methanol/chloroform (4/1, v/v) and molecular species were quantified using electrospray ionization mass spectrometry on a triple quadrupole instrument (Thermo Fisher Quantum Ultra) employing shotgun lipidomics methodologies. Both phosphatidylcholine and lysophosphatidylcholine molecular species were quantified as lithiated adducts in the positive ion mode using neutral loss scanning for 59.1 amu (collision energy = -28eV). Phosphatidylethanolamine molecular species were quantified in the negative ion survey scan mode following their derivatiza­tion to fMOC derivatives and in negative ion mode employing NL scanning for 222.2 amu (collision energy = 30 eV). Individual molecular species were quantified by comparing the ion intensities of the individual molecular species to that of the lipid class internal standard with additional corrections for type I and type II ^13^C isotope effects. Using both survey scan negative and positive ion mode additional differences in outer membrane phospholipid composition were assessed using first survey scan mode and then collisionally-assisted dissociation analysis to identify novel lipid species.

***Micro-computed tomography.*** Whole body X-ray microCT (microCT 35, Scanco Medical, Wayne, PA; X-ray tube potential 70kVp, integration time 300 ms, X-ray intensity 114 µA, isotropic voxel size 36 µm, frame averaging 1, 500 projections, medium resolution scan) was conducted on WT and G609G mice for gross examination. Then, the tibiae were dissected, cleaned of surrounding soft tissue, and scanned at higher resultion (isotropic voxel size 6 µm, 1000 projections, high resolution scan). Cortical analysis of 100 slices (0.6mm) centered about the midpoint of each tibia was conducted (threshold: 250 per mille). The following parameters were quantified: total area (TA), total bone area (BA), cortical thickness (Ct.Th, TRI method), bone mineral density (BMD), and tissue mineral density (TMD) (n=3/group).

***Reagents table S1***

| REAGENT or RESOURCE | SOURCE | IDENTIFIER |
| --- | --- | --- |
| Antibodies | | |
| Lamin A (1:1000) | Abcam | Cat#ab26300 |
| β-Tubulin (1:2000) | Sigma Aldrich | Cat#T8238 |
| Cathepsin L (1:2000) | Leinco | Cat#C1391 |
| LC3 (1:1000) | Novus | Cat#NB100-2220 |
| Chemicals, Peptides, and Recombinant Proteins | | |
| glucose | Sigma-Aldrich, St Louis |  |
| insulin |  |  |
| pyruvate |  |  |
| Critical Commercial Assays | | |
| qScript™ cDNA Synthesis Kit | Quanta biosciences |  |
| SYBR SELECT MASTER MIX | Life Technologies |  |
| Experimental Models: Organisms/Strains | | |
| Mouse: Lmna^G609G/G609G^ /Strain: C57BL/6 | Lopez-Otin laboratory (Universidad de Oviedo) | |
| Oligonucleotides | | |
| Primers: *Trim63* (mouse) Forward: GGACTACTTTACTCTGGACTTAGAAC  Reverse: CAGCCTCCTCTTCTGTAAACTC | | |
| Primers: *Pax7* (mouse) Forward: GCTACCAGTACAGCCAGTATG  Reverse: GTAGGCTTGTCCCGTTTCC | | |
| Primers: *Mhc* (mouse) Forward: CCAGGGGCAAACAGGCATTCACT  Reverse: CTTCCACTGGGCCACTTCACTGTT | | |
| Primers: *Mstn* (mouse) Forward: CTAAATGAGGGCAGTGAGAGAG  Reverse: CTATTCTGGAGTACCTCGTGTTT | | |
| Software and Algorithms | | |
| Wheel running data | Clocklab software (Actimetrics, Evanston, IL) | |
| Continuous monitoring of food consumption | BiodaQ 2.3 software, (Research Diets Inc., New Brunswick, NJ) | |
| Statistical analyses | GraphPad Software, La Jolla, CA | |
| Other | | |
| X-ray microCT | microCT 35 (Scanco Medical, Wayne, PA) | |
| electrospray ionization mass spectrometry on a triple quadrupole instrument | Thermo Fisher Quantum Ultra | |
| glucometer | OneTouch Ultra®2 glucometer | |
| continuous monitoring of food consumption | BiodaQ 2.3 (Research Diets Inc., New Brunswick, NJ) | |
| NMR | Bruker BioSpinLF50 | |

**Supplementary figures:**

**
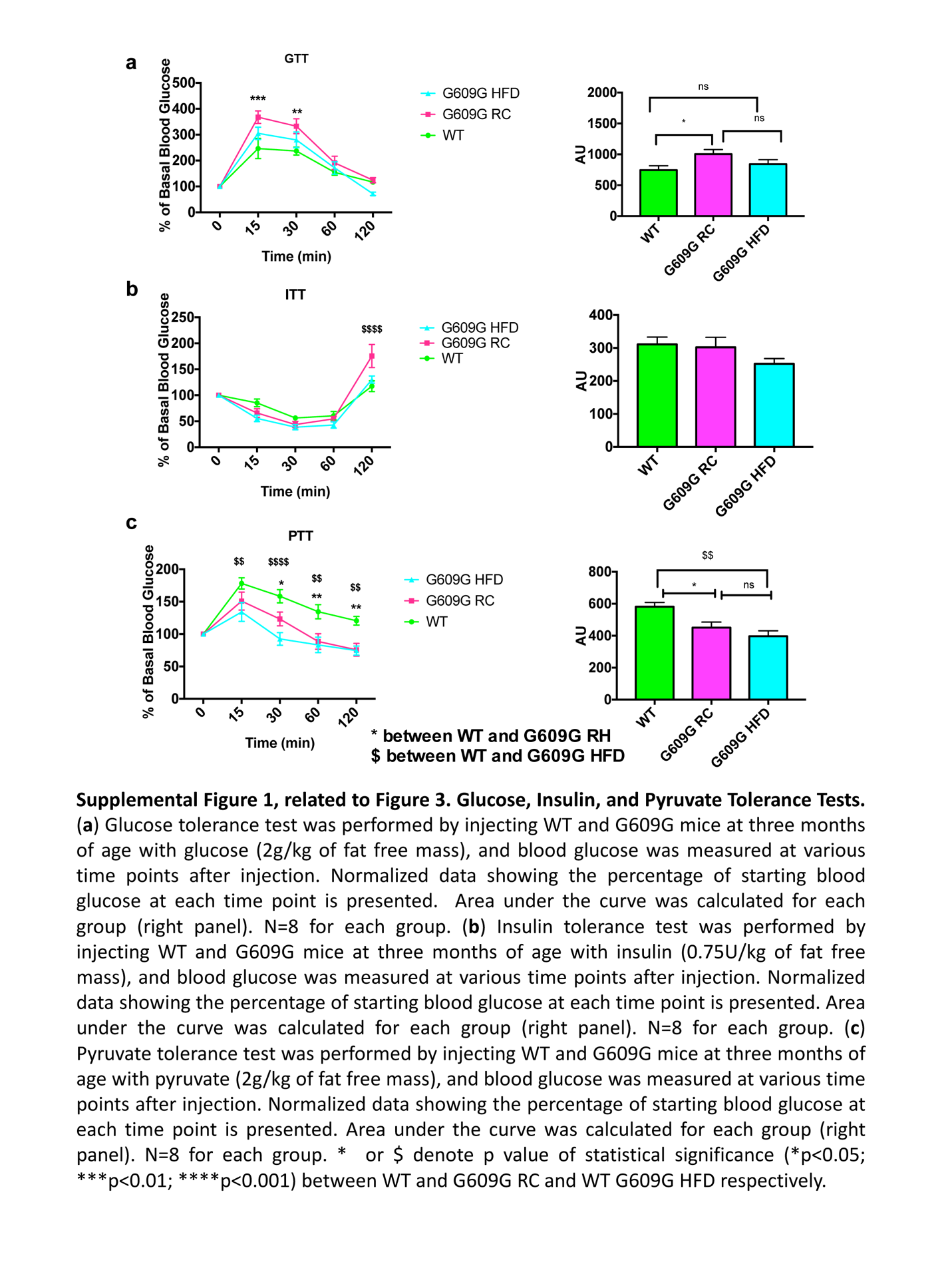
**

**Fig. S1. Glucose, insulin, and pyruvate tolerance tests.** (**a**) Glucose tolerance test was performed by injecting WT and G609G mice at three months of age with glucose (2g/kg of fat free mass), and blood glucose was measured at various time points after injection. Normalized data showing the percentage of starting blood glucose at each time point is presented. Area under the curve was calculated for each group (right panel). N=8 for each group. (**b**) Insulin tolerance test was performed by injecting WT and G609G mice at three months of age with insulin (0.75U/kg of fat free mass), and blood glucose was measured at various time points after injection. Normalized data showing the percentage of starting blood glucose at each time point is presented. Area under the curve was calculated for each group (right panel). N=8 for each group. (**c**) Pyruvate tolerance test was performed by injecting WT and G609G mice at three months of age with pyruvate (2g/kg of fat free mass), and blood glucose was measured at various time points after injection. Normalized data showing the percentage of starting blood glucose at each time point is presented. Area under the curve was calculated for each group (right panel). N=8 for each group. * or $ denote p value of statistical significance (*p<0.05; ***p<0.01; ****p<0.001) between WT and G609G RC and WT G609G HFD respectively.


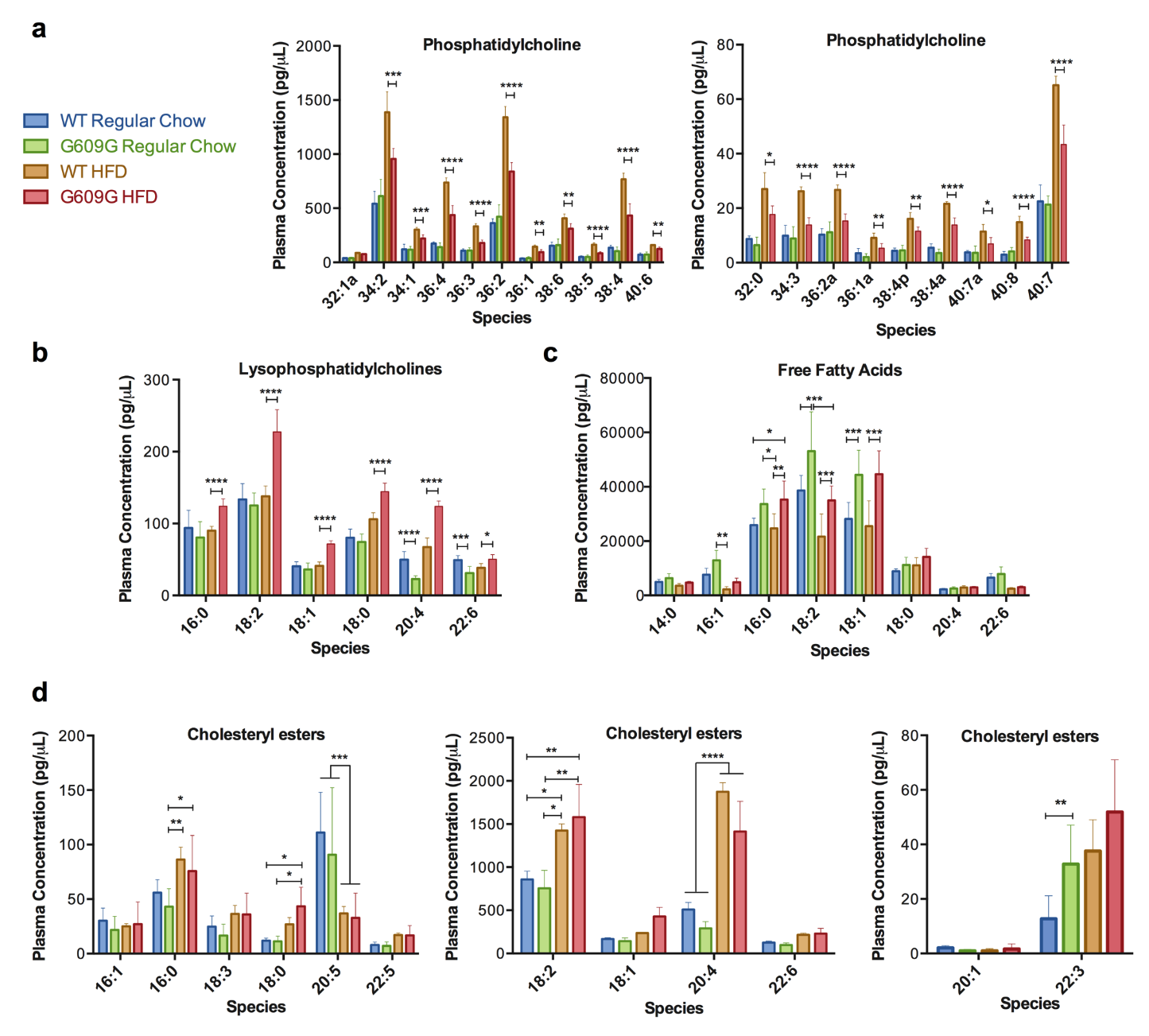


**Fig S2. Lipidomic studies in RC- and HFD-fed WT and G609G mice.** Concentrations of various species of phosphatidylcholines (a), lysophosphatidylcholines (b), free fatty acids (**c**), and cholesteryl esters (**d**) in mouse plasma, as determined by mass spectrometry in both RC- and HFD-fed WT and G609G mice.


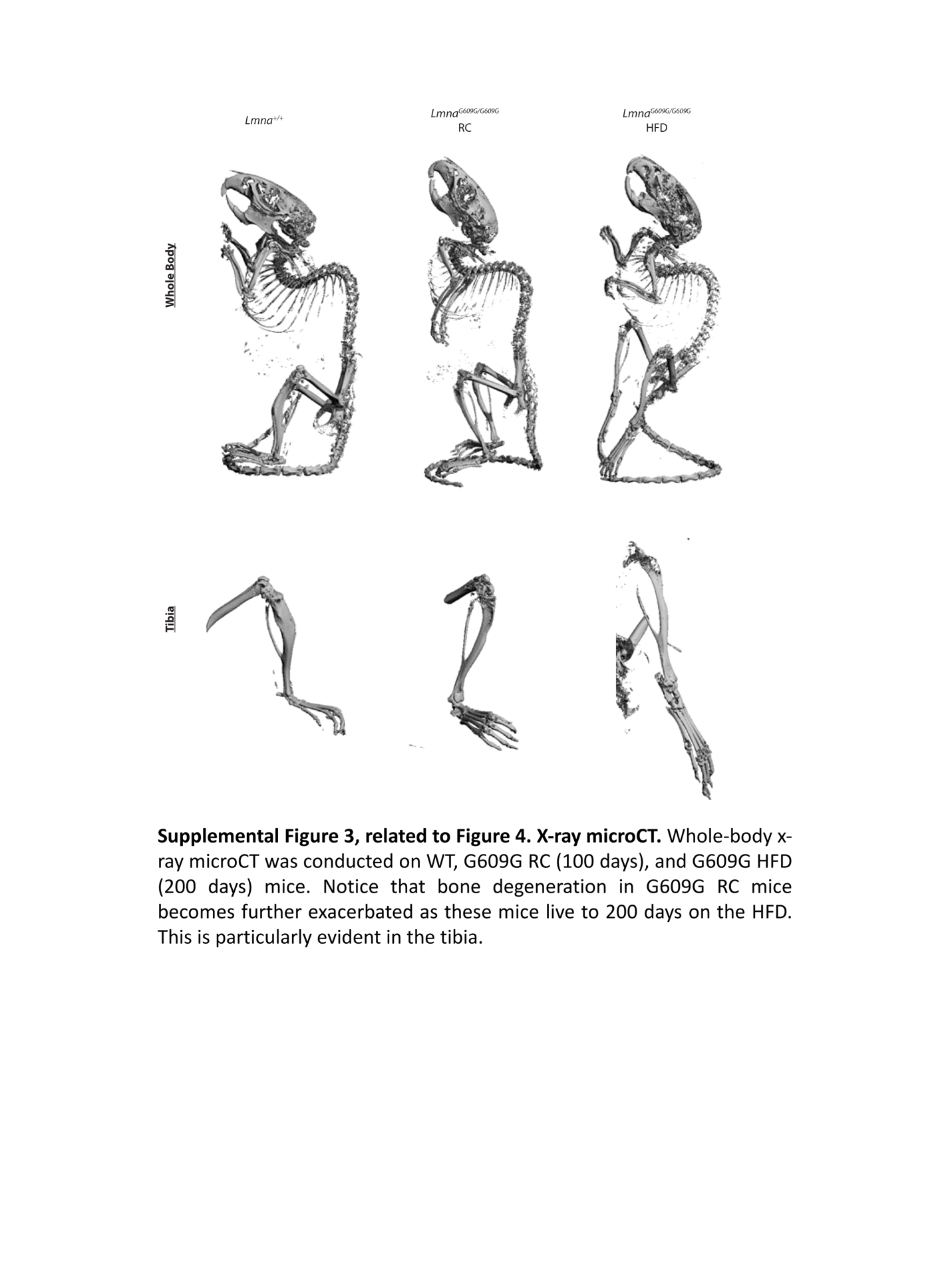


**Fig S3. X-ray microCT.** Whole-body x-ray microCT was conducted on WT, G609G RC (100 days), and G609G HFD (200 days) mice. Notice that bone degeneration in G609G RC mice becomes further exacerbated as these mice live to 200 days on the HFD. This is particularly evident in the tibia.


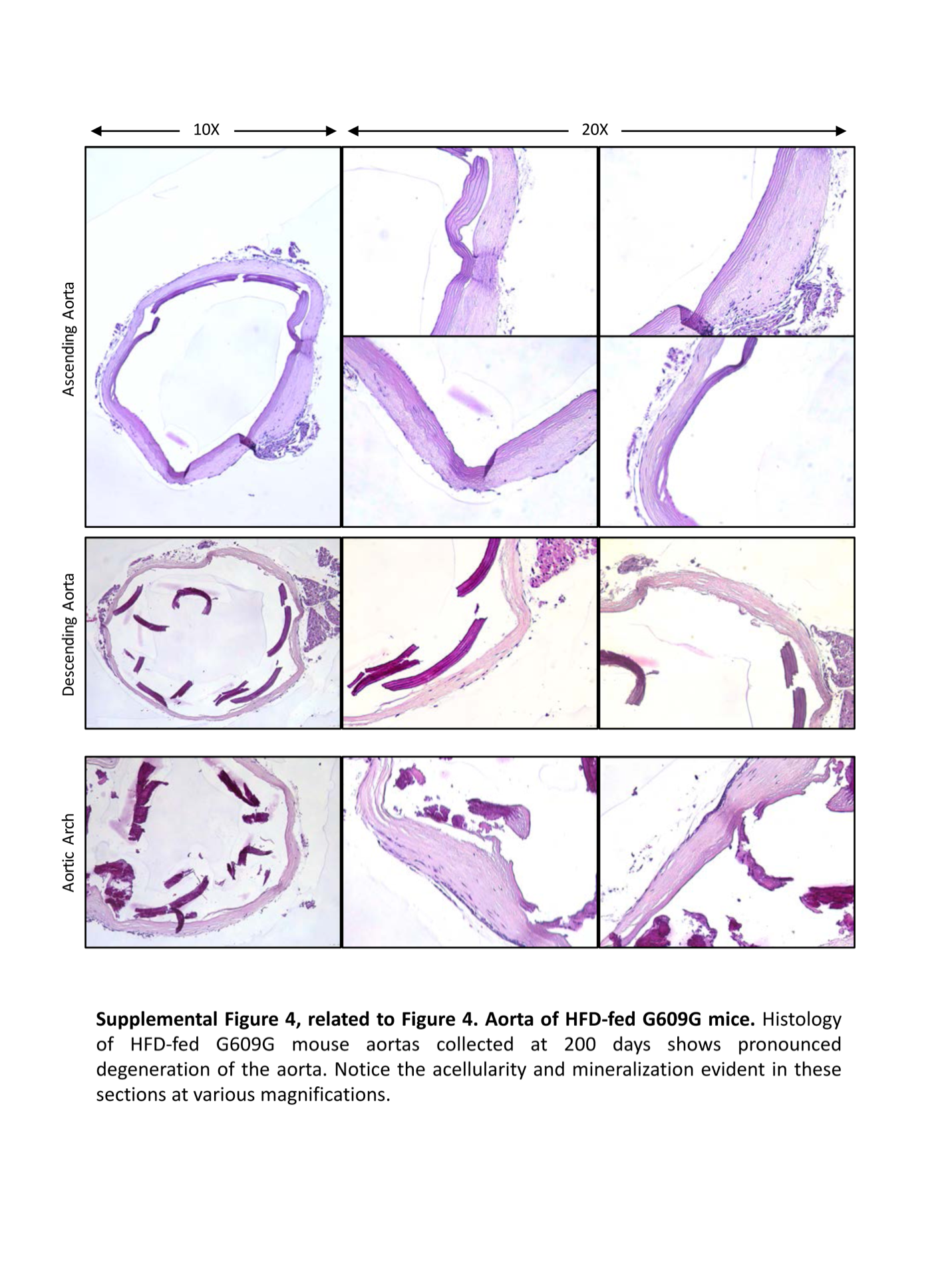


**Fig S4. Aortas of HFD-fed G609G mice.** Histology of HFD-fed G609G mouse aortas collected at 200 days shows pronounced degeneration of the aorta. Notice the acellularity and mineralization evident in these sections at various magnifications.

**Other supplemental files:**

Movie S1. Improvement of phenotypes of G609G mice on HFD. WT and G609G mice were maintained in RC until the G609G mice began to lose weight and showed pronounced lethargy, indicating that they were approaching death. Then, mice were switched to HFD, which resulted in an immediate improvement of the G609G mice. This video shows how the G609G mouse, although smaller in size, appears very active, which was a dramatic improvement from before the diet switch.

Movie S2. **Different feeding behavior between WT and G609G mice fed RC**. Shows feeding behavior of WT mice (first cage shown), and G609G mice (second cage shown). Note how G609G mice are frantically searching food while awake.
